# Radiotherapy for early and advanced stages Follicular Lymphoma

**DOI:** 10.6061/clinics/2021/e2059

**Published:** 2021-01-11

**Authors:** Geovanne Pedro Mauro, Carolina Trindade Mello Medici, Lucas Coelho Casimiro, Eduardo Weltman

**Affiliations:** I Departamento de Radiologia e Oncologia, Faculdade de Medicina (FMUSP), Universidade de Sao Paulo, Sao Paulo, SP, BR; II Faculdade de Medicina, Universidade Nove de Julho (UNINOVE), Sao Paulo, SP, BR; III Departamento de Radioterapia, Hospital do Cancer de Barretos, Porto Velho, RO, BR; IV Departamento de Radioterapia, Instituto do Cancer do Estado de Sao Paulo (ICESP), Faculdade de Medicina (FMUSP), Universidade de Sao Paulo, Sao Paulo, SP, BR; V Departamento de Radioterapia, Hospital Israelita Albert Einstein, Sao Paulo, SP, BR

**Keywords:** Radiotherapy, Follicular Lymphoma, Indolent Lymphoma

## Abstract

**OBJECTIVES:**

To evaluate the results of radiotherapy (RT) for follicular lymphoma (FL) under different management scenarios.

**METHODS:**

We retrospectively assessed consecutive patients with FL who had undergone irradiation between 2010 and 2018. All patients had biopsy-proven FL and were positron emission tomography-staged, although some (35.3%) were reassessed with computed tomography after treatment alone. Rituximab was only available to FL patients after 2016.

**RESULTS:**

Thirty-four patients were selected, with a mean age at diagnosis of 61.6 years (34-89 years). The median follow-up duration was 49.4 months. Most patients were female (58.8%) and showed good performance on the Eastern Cooperative Oncology Group (ECOG) scale (ECOG 0-55.9%). The mean overall survival (OS) and progression-free survival were 48.7 and 33.6 months, respectively, with four deaths reported. OS rates at 2 and 3 years were 94.1% and 91.2%, respectively. Four patients showed transformation into aggressive lymphomas and underwent rituximab-based systemic treatment. Transformation-free survival was 47.8 months, and all patients with transformed disease were alive at assessment. Five patients had in-field relapse, all of them also relapsed elsewhere, and the mean relapse-free survival time was 40.3 months. No median end points were reached on assessment.

**CONCLUSION:**

FL is an indolent disease. Our findings show good outcomes for patients treated with radiation, with a low transformation rate and excellent management of relapsed disease. RT is an important part of these results.

## INTRODUCTION

Follicular lymphoma (FL) is an indolent B-cell lymphoma that is often treated with radiotherapy (RT) alone at early stages and with a combination of immunochemotherapy and RT at advanced-stage disease (1).

Although the remission rate has been stable and high for a long time, typical management has changed over the years.

The most important development in the management of FLs is the introduction of rituximab. The introduction of rituximab, one of the most efficient oncological drugs, has changed how non-Hodgkin lymphomas have been treated since the publication of the MabThera International Trial (2). For indolent and low-grade lymphomas, notably FL, the drug also has an impact on the remission rate (3), also shown in earlier stages (4).

Prognostic scores have also improved. The Follicular Lymphoma International Prognostic Index 2 (FLIPI-2) was validated in 2009 (5). This prognostic index, which is used worldwide and in our institution, is one of the main tools used to predict prognosis and to correctly assess patients with FL. Although FL is a very indolent disease and seldom causes death, its management can be difficult if patients are not correctly assessed and necessary treatment is not performed.

The way how this disease is staged also has changed recently. Positron emission tomography with ^18^F-fluorodeoxyglucose (^18^F-FDG) plays an important role in staging non-Hodgkin lymphomas. For FL, this was assessed in prospective data (6) and correctly correlated with survival, although it has a low accuracy in detecting bone marrow involvement (7).

Treatment for the different stages has also evolved. Limited-stage FL has been treated with involved-field RT for decades, with good outcomes (8). Recent studies have demonstrated improved results with rituximab, as previously stated, and also with cytotoxic chemotherapy (9,10). For stage III disease, the largest report of treatment is still from radiotherapy alone (11), but combination of radiotherapy, chemotherapy and rituximab is current practice (12). Limited data are available on stage IV disease, and treatment approaches can vary from observation to combination treatments, depending on the patients’ performance and prognoses (13).

Death because of FL progression is rare, but a far more common concern is its transformation to aggressive lymphomas. Different publications have reported an approximately 10% chance of transformation of FL to aggressive histologies (14). This event can change the natural history of disease progression and is an important cause of events in this population.

This study aims to report our single-institutional experience with FL, to describe our current treatment and management approaches and results in a universal public system as a university hospital in the setting of FL staged with ^18^F-FDG PET/CT, and to describe our results.

## MATERIAL AND METHODS

All patients diagnosed as having non-Hodgkin lymphoma and treated with RT between 2010 and 2018 were retrospectively assessed. Patients treated with chemotherapy alone or those who were only observed were not selected. Only patients with FL confirmed in biopsied tissue were included. Patients also must have at least 6-months of follow up or followed until death. Patients were staged according to the Ann Arbor staging system, and therapy response was assessed using the Lugano criteria (15). RT was used either as the only prescribed treatment or as a consolidative treatment after chemotherapy and immunotherapy.

Overall survival (OS), progression-free survival (PFS), transformation-free survival (TFS), and survival free of in-field progression (SFIFP) were evaluated from the diagnosis date. Second progression-free survival (PFS2) was assessed as the period from the date the first progression was recorded to the date of second progression or death. Toxicity related to RT was evaluated according to the National Cancer Institute criteria (Common Terminology Criteria for Adverse Events v4.0 [CTCAE]) (16).

The Kaplan-Meier method was used for survival analysis. Univariate analysis was performed for each variable. Multivariate analysis was not performed because of the limited number of events. The significance level was set at 5% (*p*<0.05).

### Ethics

This research was submitted to a local ethics committee, and approval was obtained in June 2018. Ethics committee authorization was obtained from the local ethics committee in accordance with the Brazilian law and the Declaration of Helsinki.

The requirement for patient consent was waived by the ethics committee because of the retrospective nature of the study.

## RESULTS

During the study period, 34 patients with FL were treated with radiation at our institution. Patients with an uncertain histology or in whom histological analysis could not be performed were excluded. The median follow-up duration was 49.4 months. Most patients were female (58.8%), and the mean age at diagnosis was 61.7 years. The performance status was assessed using the Eastern Cooperative Oncology Group scale, and most patients scored 0-1, whereas only seven (20.6%) had a compromised performance at diagnosis. Most patients had low-grade disease (79.4%). Stages were diverse, and almost half (47.1%) were diagnosed as having limited disease (stages I and II). Seven patients (20.6%) had extranodal disease at diagnosis.

The FLIPI-2 prognostic scale was used to establish the prognosis. Although all patients had already been scored on their charts, we retrospectively reviewed all examinations to correctly determine the FLIPI-2 score at diagnosis and to prevent bias. Thereafter, the most common FLIPI-2 class was intermediate risk (70.6%). Patient characteristics are described in Table 1.

**Figure 1:**
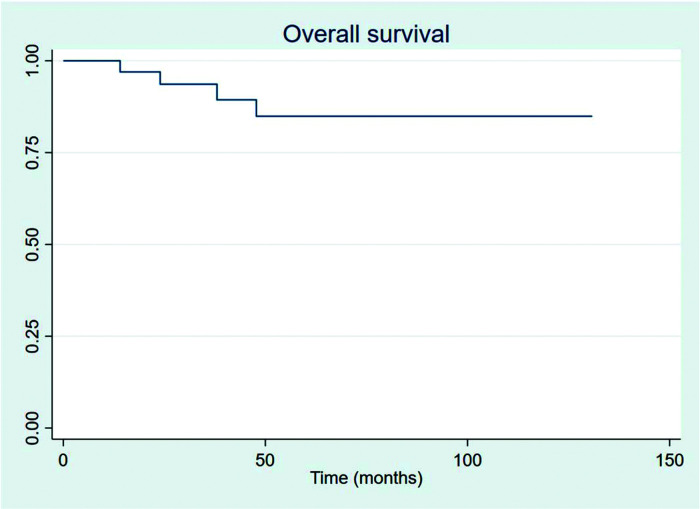
Overall survival.

**Figure 5:**
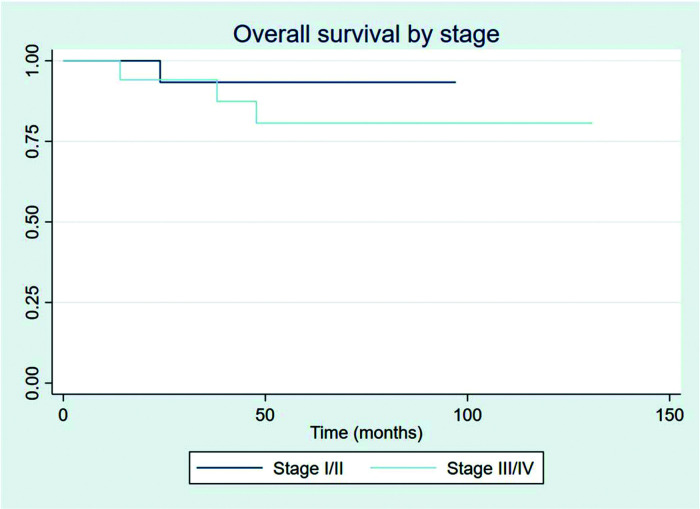
Overall survival by stage.

The outcomes of this study reflect previously published data. The mean OS, TFS, SFIFP, and PFS were 48.7, 47.8, 40.3, and 33.6 months, respectively, and are presented in Figures 1-4, respectively. Figure 5 shows OS by stage. Only PFS reached a median value of 69.3 months. The OS rates at 2 and 3 years were 94.1% and 91.2%, respectively.

**Figure 6:**
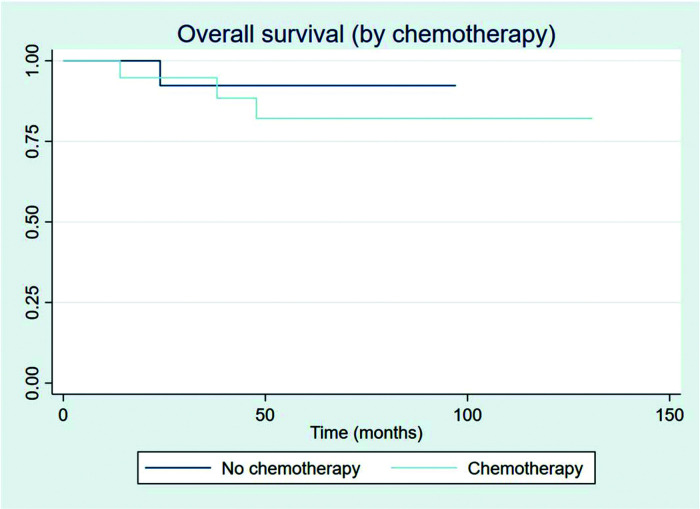
Overall survival by chemotherapy group.

Patients showed a good response rate to chemotherapy. Of the 20 patients who received chemotherapy, 8 (40%) showed a complete response, and 11 (55%) a partial response. Only one patient showed progression after first-line chemotherapy and was treated with 36 Gy radiation to the site of disease progression thereafter; this patient did not have another event and is currently disease-free. Two patients had stage IIXB disease with large abdominal masses. All other patients were diagnosed as having advance-stage disease. As stated previously, all patients receive RT as part of their treatment. After chemotherapy, the outcomes for limited and advanced-stage disease did not differ, as can be seen in Figure 6 (*p*=0.608). Patients with extranodal disease were often more likely to have advanced-stage disease (6/9) and received chemotherapy. The other three patients were stage IIXE with cervical presentations and received radiation to their nodal and extranodal disease sites.

Rituximab is often used as part of the treatment regimen. Half (10) of the patients who received chemotherapy also received rituximab. At our institution, we did not have the drug available for earlier stages of RT alone, but it was available for advanced stages, concurrent with chemotherapy. Primary treatment is indicated for patients on the basis of the GELF/NBLI criteria (17), and prognosis is stated according to FLIPI-2. There was no statistical impact of immunotherapy on univariate analysis, but this is mainly because of the very low frequency of events in our sample.

**Figure 7:**
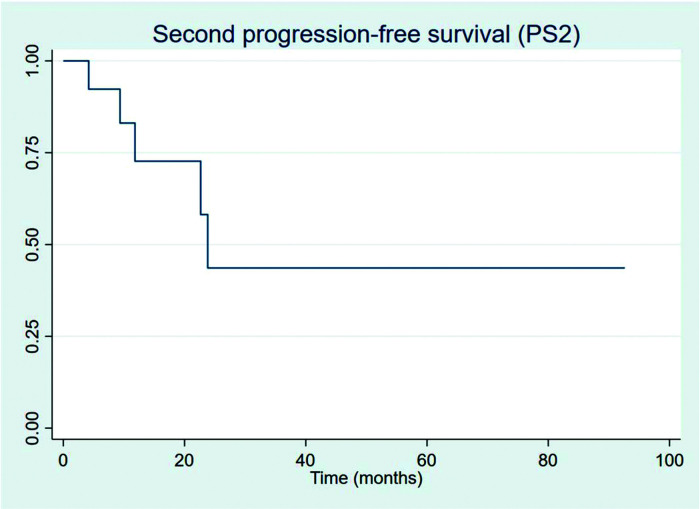
Second progression-free survival (from the date of first progression).

All 13 progressions were treated with second-line chemotherapy, and only 5 (38.5%) progressed subsequently. For the patients who PFS2 could be measured, one patient received third-line chemotherapy and is still alive by last evaluation and one patient died without receiving any further chemotherapy treatment; all other three other patients are currently under watchful-waiting. Figure 7 shows PFS2 recorded from the date of first progression.

Four patients (11.8%) who were diagnosed as having transformed disease were treated with second-line chemotherapy (CHOP) plus rituximab. No other patient presented any other event.

In-field progression (IFP) is also rare. IFP was defined as disease progression within previously irradiated sites. Five patients (14.7%) presented progression within the radiation field, which also progressed outside the radiation field. All patients with IFP had advanced disease stages. Nevertheless, all patients with IFP received second-line chemotherapy and had no other events. Table 2 shows the RT characteristics.

Mortality was low and preceded by other events. An important cause of death in our group was secondary malignances, which occurred in two patients. One patient with FL stage IVXEB and an abdominal bulky disease developed stage IV rectal adenocarcinoma that began marginally to the prior radiation field. A second patient with stage IIB FL treated with exclusive RT was subsequently diagnosed as having squamous carcinoma of the pelvis outside the radiation field. Both patients died because of secondary malignances. Another cause of death was progression. One patient showed progression, could not receive second-line chemotherapy because of low performance, and eventually died. The last patient died while receiving third-line chemotherapy for FL. Table 3 summarizes the toxicities associated with treatment.

## DISCUSSION

Our study has some limitations that should be addressed. It includes a small, single-institution sample that was retrospectively assessed. Another important aspect of our cohort that should be highlighted is that FL is a very indolent disease and prospective studies in this setting are usually performed for longer follow-up periods. Although some of our patients were under follow-up for more than 10 years, the median follow-up period was still short. Another point of consideration is the staging procedure. All patients were staged using PET, but only 23.5% of the responses were assessed using this method. None of the end points reached the median values, as is common with very curable diseases in the early stages.

This study shows the results of radiation as part of FL management. All patients underwent radiation as the first-line treatment for their disease. Our study shows good results in both early and advanced stages, with most patients being alive at assessment. Progression, although not unusual, can be managed by immunochemotherapy or RT, often a combination of both. Further, transformation is also quite rare and manageable.

Our findings show good treatment results for FL, notably in the early-stage setting. Brady et al. (VI) reported on a multi-institutional cohort of early-stage FL with similar progression and survival outcomes over the same follow-up period, which allows us to believe that our indications and protocols are consistent with international consensus.

Findings on RT for advanced-stage FL are neither as common nor as current as those for early disease. This study presents new data on RT as a consolidative treatment for advanced FL, with a large group of patients, which is scarce in the literature. When chemotherapy and rituximab are added to radiation in this setting, survival results are similar to those of early disease and therefore when combination treatment is favored, results in advanced stages can be enhanced to become similar to those at earlier stages.

Toxicities were rare with RT. As shown in Table 2, RT for FL is often well tolerated, and late toxicities are seldom seen. Secondary malignances were also observed and were rare, although life-threatening. It is important to keep these in mind when treatment with radiation is favored, as these can be late complications. However, not all secondary malignances can be traced back to RT. These results demonstrate that radiation is an option with few side effects and a good cost-benefit ratio.

## CONCLUSION

The outcomes of RT for patients with FL are good, mainly in combination with systemic treatment.

## AUTHOR CONTRIBUTIONS

Medici C and Mauro G were responsible for study design and ethics committee approval. Medici C and Casimiro L were responsible for data collection and the project’s final draft. Mauro G was responsible for the statistical analysis. Mauro G, Medici C, and Casimiro L were responsible for writing the manuscript. Weltman E was responsible for overall orientation and manuscript review.

## Figures and Tables

**Figure 2 f02:**
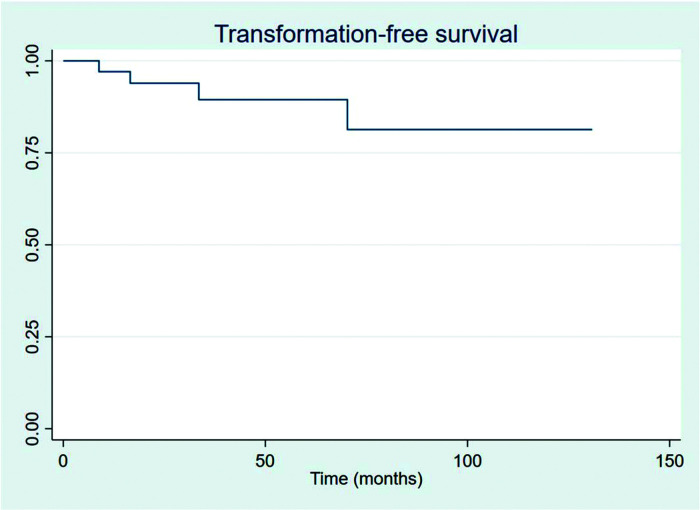
Transformation-free survival.

**Figure 3 f03:**
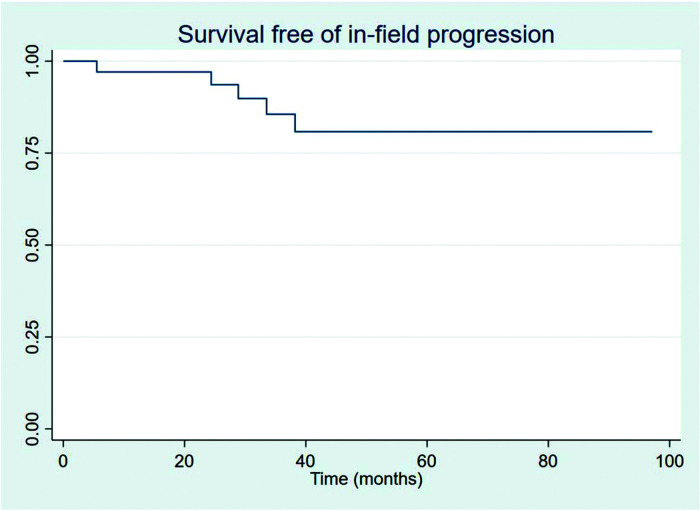
Survival free of in-field progression.

**Figure 4:**
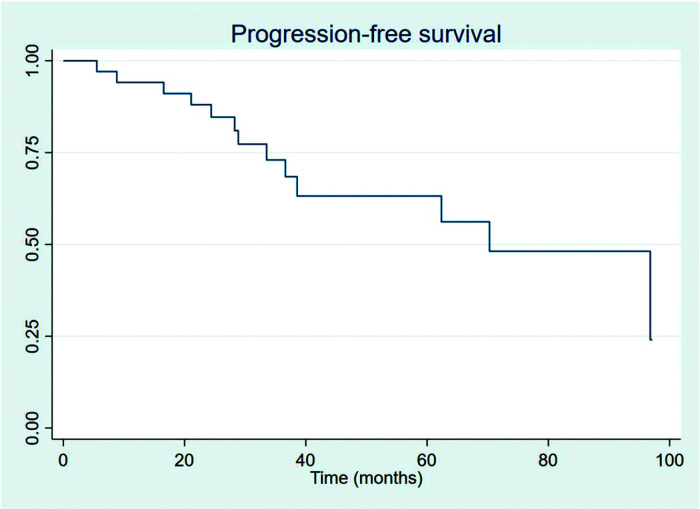
Progression-free survival.

**Table 1:** Patient characteristics.

	Number (n)	(%)
Sex		
Male	14	41.2
Female	20	58.8
ECOG performance status		
0	19	55.9
1	8	23.5
>1	7	20.6
Histological grade		
1-2	27	79.4
3	7	20.6
Stage		
I-II (localized)	16	47.1
III-IV (advanced)	18	52.9
FLIPI-2		
Low risk	6	17.6
Intermediate risk	24	70.6
High risk	4	11.8
Extranodal involvement		
Yes	7	20.6
No	27	79.4
Bone marrow involvement		
Yes	6	17.6
No	28	82.4
Bulky disease (>7 cm)		
Yes	21	61.8
No	13	38.2
B symptoms		
Yes	8	23.5
No	26	76.5
Lymph node size		
Mean	7.28 cm	
Lower	0.5 cm	
Maximum	21.5 cm	

**Table 2:** Radiotherapy and treatment.

	Number (n)	(%)
RT dose		
<24 Gy	7	20.6
30 Gy	14	41.2
36 Gy	12	35.3
40 Gy	1	2.9
RT technique		
Involved-field RT (IF RT)	14	41.2
Extended-field RT (EB RT)	2	5.9
Only bulky and partial response	18	52.9
Chemotherapy		
CHOP	12	35.3
CHO MP	2	5.9
Other	6	17.6
No chemotherapy	14	41.2
Rituximab		
Yes	10	29.5
No	24	70.5
Response to chemotherapy		
No chemotherapyDisease progression	14	41.2
Disease progression	1	2.9
Partial response	11	32.4
Complete response	8	23.5
Response assessment method		
PET	8	23.5
CT	12	35.3
No chemotherapy	14	41.2

**Table 3:** Toxicities.

Toxicity	Grade	Number	(%)
To chemotherapy	<3	7	33.3
	3-4	13	61.9
	No info	1	0.4
	No chemo	13	-
	No rituximab	10	-
Acute to radiotherapy	0-1	26	76.4
	>1	8	23.5

Type of toxicity	Type	Number	(%)
Most common side effect to RT	GI	17	50.0
	Skin	4	11.7
	No toxicity	13	61.9
